# Spontaneous Intracranial Hypotension in a Patient with Systemic Lupus Erythematosus and End-Stage Renal Failure: A Case Report and a Literature Review

**DOI:** 10.3390/brainsci15030296

**Published:** 2025-03-12

**Authors:** Konstantinos Paterakis, Alexandros Brotis, Adamantios Kalogeras, Maria Karagianni, Theodosios Spiliotopoulos, Christina Arvaniti, Argiro Petsiti, Marianna Vlychou, Efthimios Dardiotis, Eleni Arnaoutoglou, Kostas N. Fountas

**Affiliations:** 1 Department of Neurosurgery, University Hospital of Larissa, School of Medicine, University of Thessaly, 41500 Larissa, Greece; kpaterakis@yahoo.com (K.P.); kalogadam@gmail.com (A.K.); maria.karagianni.1994@gmail.com (M.K.); spilteo90@gmail.com (T.S.); arvanitixristina@hotmail.com (C.A.); fountas@uth.gr (K.N.F.); 2 Department of Anesthesiology, University Hospital of Larissa, 41334 Larissa, Greece; apetsiti@yahoo.gr (A.P.); earnaout@gmail.com (E.A.); 3 Department of Radiology, University General Hospital of Larissa, 41100 Larissa, Greece; mvlychou@uth.gr; 4 Department of Neurology, University Hospital of Larissa, School of Medicine, University of Thessaly, 41500 Larissa, Greece; edar@med.uth.gr

**Keywords:** spontaneous intracranial hypotension, end-stage renal failure, systemic lupus erythematosus, epidural blood patches, cranial subdural collections, surgical evacuation

## Abstract

***Background and Objectives***: End-stage renal failure (ESRF) patients are at an increased risk of various neurological complications, particularly after hemodialysis. The current case report describes a rare presentation of spontaneous intracranial hypotension (SIH) in a patient with ESRF caused by systemic lupus erythematosus (SLE). ***Methods****:* We present our case report. We also performed a systematic literature search in PubMed, Scopus, and Dimensions for the current literature review. ***Results***: A total of 296 unique articles were identified, and their full text was retrieved. However, only one case report was relevant to our study and is summarized thereunder. The treatment approach involved high-dose intravenous steroids, surgical evacuation of the cranial subdural collections, and epidural blood patches to seal the presumed dural defect. ***Conclusions***: This case report describes a rare presentation of SIH in a young patient with ESRF due to SLE. Diagnostic imaging revealed extensive subdural and epidural fluid collections in the brain and spinal cord, respectively, along with a few T2 FLAIR hyperintensities noted in the right thalamus, left cerebellar hemisphere, and right occipital gyrus that subsequently resolved. The treatment approach involved high-dose intravenous steroids, surgical evacuation of the cranial subdural collections, and epidural blood patches to seal the presumed dural defect.

## 1. Introduction

End-stage renal failure (ESRF) patients are at an increased risk of various neurological complications, particularly after hemodialysis [1,2]. These complications can range from cerebrovascular events to encephalopathy and are often related to the complex interplay between renal dysfunction, cardiovascular factors, and impaired autoregulation [1,2]. The current case report describes a rare presentation of spontaneous intracranial hypotension (SIH) in a patient with ESRF caused by systemic lupus erythematosus (SLE). It discusses the potential underlying mechanisms and management considerations. It might be of interest to clinicians involved in managing chronic renal failure, including nephrologists, as well as with other inter-related disciplines, such as rheumatologists, neurologists, neurosurgeons, and anesthesiologists.

## 2. Case Report

**Figure 1:**
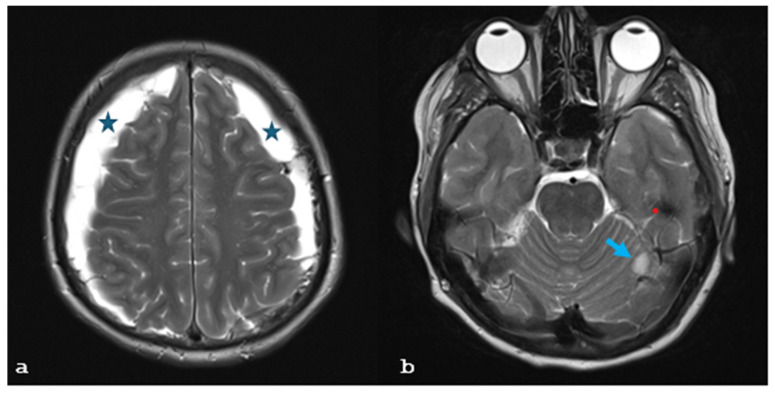

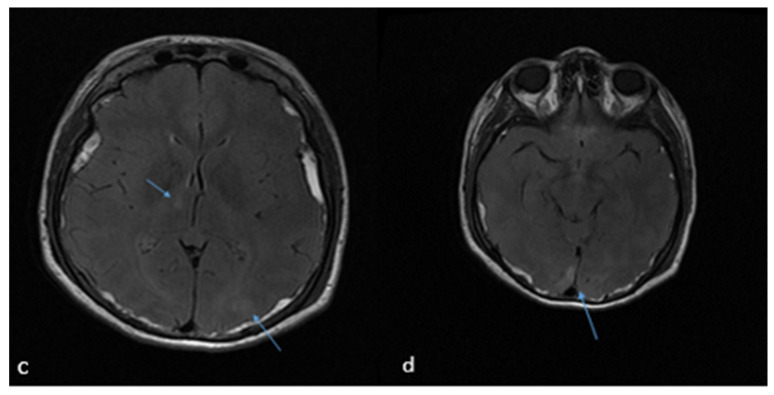
(**a**–**d**): Brain MRI T2-weighted images show (**a**) bilateral subdural collections (asterisks) larger than 1.2 cm and (**b**) a lesion (arrow) with increased signal abutting the left cerebellar peduncle and a lesion with hypointense signal in the left temporal lobe (red dot). (**c**,**d**) T2 FLAIR hyperintensities (arrows) at the right thalamus, left occipital lobe, and right occipital lobe.

**Figure 2:**
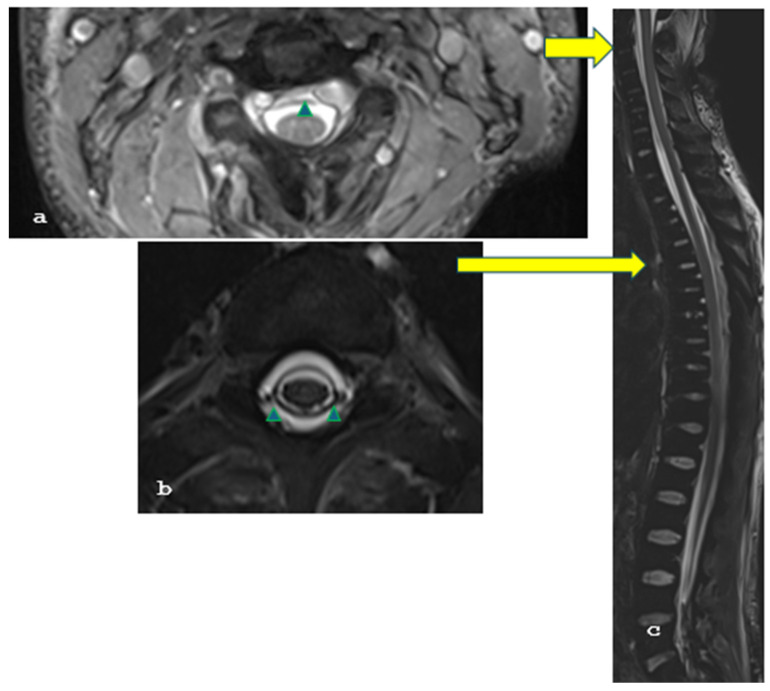
(**a**–**c**): Axial and sagittal T2-weighted MR images reveal circumferential CSF collections (green arrowheads) anterior to the cervical and on all sides of the thoracic spinal cord (yellow arrows), showing these collections on a sagittal image.

A 26-year-old female patient with a longstanding 14-year history of SLE and ESRF presented with a 2-week history of severe headache, which was exacerbated when transitioning from a sitting to a standing position, nausea, vomiting, and blurred vision. On clinical examination, the patient was alert and oriented, with a Glasgow Coma Scale score of 15/15, and exhibited mild cervical dystonia and paresis of the left abducens nerve without any other focal neurological deficits. Brain magnetic resonance imaging (MRI), and particularly T2-weighted images, revealed extensive bilateral subdural collections larger than 1.2 cm, pachymeningeal enhancement, and ventricle narrowing, strongly suggestive of intracranial hypotension. In addition, there were a few T2 FLAIR hyperintensities at the right thalamus, left occipital lobe, and left cerebellar peduncle (Figure 1a–c), indicative of posterior reversible encephalopathy syndrome (PRES) syndrome. Moreover, the cervicothoracic spinal T2-weighted images and the MRI demonstrated an anterior epidural cerebrospinal fluid collection measuring 2.7 mm in thickness, extending from C2 to C4, as well as a circumferential collection 3 mm thick, spanning from Th2 to Th7 (Figure 2a–c). This clinical and radiological presentation was consistent with the diagnosis of SIH, likely due to a spinal dural tear in the context of the patient’s underlying SLE.

**Figure 3:**
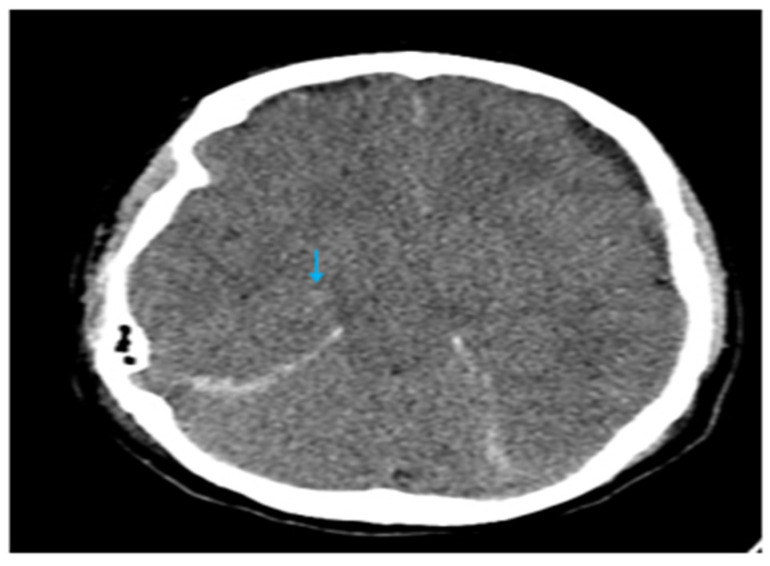
Axial CT image, which indicates subarachnoid hemorrhage and a small hyperdense focus (arrow) located at the right thalamus, indicative of PRES.

**Figure 4:**
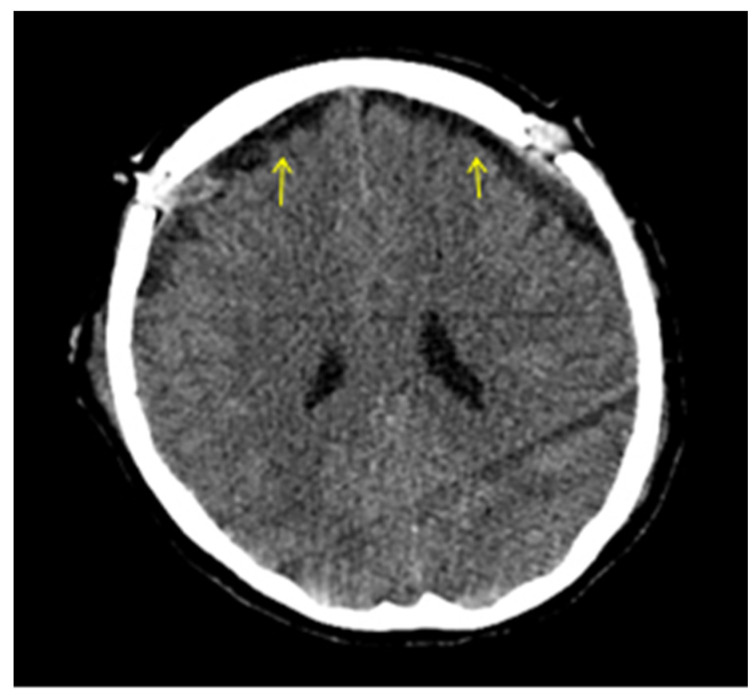
CT images immediately after surgery. The reduction in subdural collections is obvious postoperatively (yellow arrows).

It is noteworthy that while efforts were being made to arrange a session involving anesthesiologists, nephrologists, neurologists, and neurosurgeons to determine the treatment plan, the patient’s headache worsened during hemodialysis, requiring escalated doses of analgesics, including opioids. Following consultation with the nephrology department, we focused on maintaining fluid balance during the hemodialysis sessions, without implementing any additional treatments or measures prior to each session. During one such dialysis session, the patient became obtunded and unresponsive, requiring urgent intubation and mechanical ventilation (GCS 7—E1V2M4). Subsequent brain computed tomography (CT) revealed new hemorrhagic foci within the basal ganglia and periventricular white matter (Figure 3). The size of the subdural collections was practically unchanged, but there was significant brain sagging. The patient was started on high-dose intravenous methylprednisolone for supportive care. Subsequently, a 15 mL epidural blood patch was injected at the Th7–Th8 level and an additional 15 mL at the L2–L3 level under continuous neuromonitoring to seal the presumed dural defect. In the same setting, the patient underwent bilateral burr hole evacuation of the subdural collections using a closed drainage system. Two days later, the patient was successfully weaned from the ventilator and extubated. Her headaches and visual symptoms had resolved, and she was discharged to the Neurosurgery department in a stable condition after a brief observation period of a few days. The postoperative CT showed the presence of bilateral subdural collections, markedly reduced (less than 5 mm) (Figure 4).

**Figure 5:**
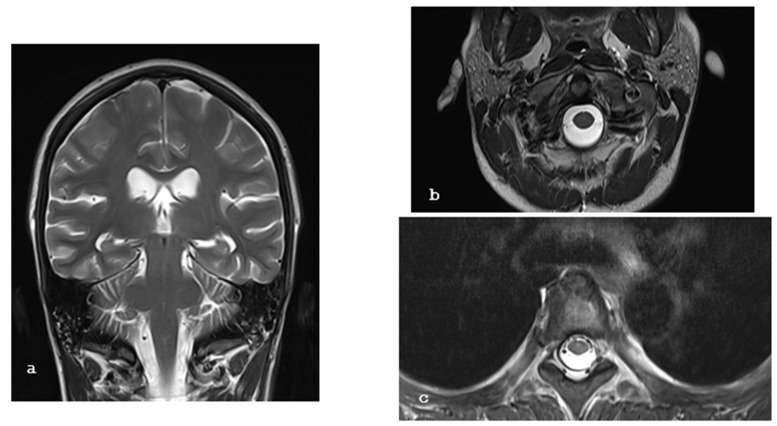
(**a**–**c**): Coronal and axial T2-weighted MR images of the brain, cervical, and thoracic spine one month postoperatively show no evidence of subdural collections or perispinal fluid accumulation.

During her hospital stay, the patient developed a fever, and blood cultures confirmed the presence of *Streptococcus pneumoniae*. Additionally, a transesophageal echocardiogram revealed a mobile mass attached to the mitral valve, indicating the development of infective endocarditis. This complication was appropriately managed with intravenous antibiotics. After sufficient intravenous and oral antibiotics, the patient was discharged home in significantly improved condition. At follow-up, the patient remained asymptomatic and resumed her normal daily activities. The subdural and epidural collections were not obvious at the last follow-up, one month after surgery (Figure 5a–c).

## 3. Literature Search

We performed a systematic literature search in PubMed, Scopus, and Dimensions for the current literature review. We focused on articles reporting cases of intracranial hypotension secondary to dural tears in patients with end-stage renal disease. There were no limitations regarding the publication language or any other temporal restriction. The search string comprised the following terms: “intracranial hypotension”, “craniospinal hypotension”, “ESRD”, “renal failure”, “hemodialysis”, and “end-stage kidney disease” (Table 1). A total of 296 unique articles were identified, and their full text was retrieved. However, only one case report was relevant to our study and is summarized thereunder [3].

## 4. Discussion

This case report describes a rare presentation of SIH in a young patient with ESRF due to SLE. The patient experienced postural headaches that worsened during hemodialysis sessions, leading to disturbances of consciousness. Diagnostic imaging revealed extensive subdural and epidural fluid collections in the brain and spinal cord, respectively, as well as a few T2 FLAIR hyperintensities with asymmetric distribution that resolved during imaging follow-up, indicative of posterior reversible encephalopathy syndrome (PRES). The treatment approach involved high-dose intravenous steroids, surgical evacuation of the cranial subdural collections, and epidural blood patches to seal the presumed dural defect.

The use of steroids in the treatment of spontaneous intracranial hypotension (SIH) presents a clinical dilemma. While successful conservative treatment is observed in fewer than a quarter of SIH cases, the potential clinical benefits of steroids have been attributed to several mechanisms, including the reduction in brain edema and inflammation, promoting fluid retention, and aiding the reabsorption of cerebrospinal fluid (CSF) from the extradural space. Despite the fact that the epidural blood patch remains the most effective treatment for SIH, the question arises whether steroids should be considered as a first-line therapy. Future studies are needed to evaluate the role of steroids in conjunction with more commonly recommended measures such as bed rest and hydration to determine their true effectiveness in managing SIH [3].

The literature search identified one relevant case report. Schievink and Maya reported the case of a 37-year-old woman with ESRF who experienced persistent lethargy and headaches during hemodialysis [4]. Imaging revealed brain sagging and presyrinx, which worsened despite treatments such as epidural blood patches and fibrin sealant injections [4]. Ultimately, surgical repair of leaking CSF and reinforcement of arachnoid cysts resulted in the complete resolution of her symptoms [4]. She underwent a successful renal transplant to prevent further CSF leaks and has remained headache-free during the 9 months of follow-up [4]. Our current case showed no evidence of a co-existing syrinx and did not require any treatment other than bilateral subdural collection drainage and blood patch. We decided to perform two epidural blood patches: one at the usual lumbar level for SIH, where no obvious dural tear was identified, and another at the thoracic level, where the largest collection of CSF was observed. This approach was intended to increase the likelihood of treatment success.

The underlying mechanisms of spontaneous intracranial hypotension (SIH) in patients with end-stage renal disease (ESRD) require further clarification. It has been hypothesized that the repetitive volume and pressure changes during hemodialysis may contribute to developing dural tears and subsequently lead to CSF leakage. Additionally, the relative hypotension experienced by ESRD patients during dialysis may further accentuate the intracranial hypotension [5]. The disturbances of cerebrovascular autoregulation and the increased susceptibility to stroke in ESRD patients may also predispose this population to intracranial hypotension and associated complications [5]. Furthermore, systemic lupus erythematosus (SLE) is recognized as a connective tissue disorder, and spontaneous intracranial hypotension (SIH) is frequently linked to abnormalities in connective tissue, such as dural ectasia [6]. It is reasonable to suggest that the connective tissue disorder associated with systemic lupus erythematosus (SLE), combined with the fluctuations in blood pressure that occur during hemodialysis in patients with end-stage renal disease (ESRD), further increases the risk of spontaneous cerebrospinal fluid (CSF) leaks.

SIH is characterized by low CSF pressure due to a CSF leak [7], while PRES, on the other hand, involves brain edema, often in the posterior regions [8]. It is typically associated with hypertension, renal disease, and certain medications, including immunosuppressants.

It is theoretically possible, though uncommon, for the physiological changes associated with spontaneous intracranial hypotension (SIH) to contribute to the development of posterior reversible encephalopathy syndrome (PRES), or vice versa. For example, severe and prolonged SIH could potentially lead to cerebral edema [9].

Patients with renal failure commonly experience neurological complications, which can stem from the uremic state or the treatment itself [5]. These complications include uremic encephalopathy, atherosclerosis, neuropathy, and myopathy, and they may not be fully responsive to dialysis [2,5]. Moreover, the dialysis process itself and kidney transplantation can also lead to neurological issues, such as dialysis headaches and posterior reversible encephalopathy syndrome (PRES) [10]. Furthermore, renal transplantation and the use of immunosuppressive drugs post-transplantation can result in additional neurological complications [2,5]. Renal disease also increases the risk of stroke, as demonstrated in a large retrospective study of over 19,000 dialysis patients [2,5,11].

Headaches, altered consciousness, seizures, and visual disturbance [10,12,13] characterize PRES. Hypertension and renal failure are key risk factors. PRES is associated with reversible subcortical edema, particularly in the parieto-occipital lobes [12,13]. The pathogenesis in renal failure may involve endothelial dysfunction, blood–brain barrier disruption, and autoregulatory failure. Immunosuppressants, especially calcineurin inhibitors, can also contribute to PRES, especially in kidney transplant patients [12,13]. A study of 18 maintenance hemodialysis patients with PRES found most episodes developed shortly after initiating dialysis, with all having resistant hypertension. Infections were common, and some patients experienced recurrence, but renal transplantation was safe despite prior PRES [14].

## 5. Limitations

This case report is characterized by several limitations. Given the rarity of this condition, the available literature is limited, with only one relevant case report identified. It is essential to enhance treatment strategies for spontaneous intracranial hypotension (SIH) in patients with end-stage renal disease (ESRD). The source of the dural defect remains speculative, and more advanced imaging techniques may be required to pinpoint the exact location of the CSF leak. It is unclear whether the burr hole evacuation of subdural collections and epidural blood patches was required, completely curative, or if additional interventions would have been necessary.

## 6. Conclusions

Neurological complications are common in patients with end-stage renal disease and can be challenging to diagnose and manage, especially in the context of dialysis. This case highlights the need for prompt recognition and treatment of idiopathic intracranial hypotension in patients with chronic kidney disease, as this condition can lead to life-threatening complications if left unmanaged. In this patient, the rapid onset of symptoms, the MRI findings, and the response to treatment confirmed the diagnosis. Clinicians should maintain a high index of suspicion for SIH in ESRD patients, particularly those undergoing hemodialysis, who present with unexplained headaches, altered consciousness, or other neurological symptoms.

## Figures and Tables

**Table 1:** Search strategy of our current literature search.

Database	Search String	Results
PubMed	(((“Chronic renal failure”) OR (“End-stage renal failure”)) OR (“hemodialysis”)) AND ((“Spontaneous intracranial hypotension”) OR (“Craniospinal hypotension”))	1
Scopus	(“Chronic renal failure” OR “end-stage renal failure” OR “hemodialysis”) AND (“Spontaneous intracranial hypotension” OR “craniospinal hypotension”) AND (LIMIT-TO (DOCTYPE, “ar”))	20
Dimensions	(“Chronic renal failure” OR “end-stage renal failure” OR “hemodialysis”) AND (“spontaneous intracranial hypotension” OR “craniospinal hypotension”); filter: article	296

## Data Availability

The original contributions presented in this study are included in the article. Further inquiries can be directed to the corresponding author.

## Abbreviations

ESRF	end-stage renal failure
SIH	spontaneous intracranial hypotension
SLE	systemic lupus erythematosus
MRI	magnetic resonance imaging
GCS	Glasgow Coma Scale
CT	computed tomography
PRES	posterior reversible encephalopathy syndrome
ESRD	end-stage renal disease
CSF	cerebrospinal fluid
